# From Resistance to Sensitivity: Insights and Implications of Biphasic Modulation of Autophagy by Sunitinib

**DOI:** 10.3389/fphar.2017.00718

**Published:** 2017-10-10

**Authors:** Amal Kamal Abdel-Aziz, Ashraf B. Abdel-Naim, Samia Shouman, Saverio Minucci, Mohamed Elgendy

**Affiliations:** ^1^Department of Experimental Oncology, European Institute of Oncology, Milan, Italy; ^2^Department of Pharmacology and Toxicology, Faculty of Pharmacy, Ain Shams University, Cairo, Egypt; ^3^Cancer Biology Department, National Cancer Institute, Cairo University, Cairo, Egypt; ^4^Department of Biosciences, University of Milan, Milan, Italy; ^5^Max F. Perutz Laboratories, Department of Microbiology and Immunobiology, University of Vienna, Vienna, Austria

**Keywords:** autophagy, cancer, Mcl-1, mTOR, resistance, Sunitinib

## Abstract

Sunitinib, a multityrosine kinase inhibitor, is currently the standard first-line therapy in metastatic renal cell carcinoma (mRCC) and is also used in treating patients with pancreatic neuroendocrine and imatinib-resistant gastrointestinal stromal tumors (GIST). Nevertheless, most patients eventually relapse secondary to intrinsic or acquired sunitinib resistance. Autophagy has been reported to contribute to both chemo-sensitivity and -resistance. However, over the last few years, controversial regulatory effects of sunitinib on autophagy have been reported. Since gaining insights into the underlying molecular insights and clinical implications is indispensible for achieving optimum therapeutic response, this minireview article sheds light on the role of a network of prosurvival signaling pathways recently identified as key mediators of sunitinib resistance with established and emerging functions as autophagy regulators. Furthermore, we underscore putative prognostic biomarkers of sunitinib responsiveness that could guide clinicians toward patient stratification and more individualized therapy. Importantly, innovative therapeutic strategies/approaches to overcome sunitinib resistance both evaluated in preclinical studies and perspective clinical trials are discussed which could ultimately be translated to better clinical outcome.

## Introduction

Following the initial breakthrough success of imatinib, the first FDA-approved tyrosine kinase inhibitor(TKI), TKIs were deemed to revolutionize cancer therapy. Nevertheless, emergence of imatinib-resistance prompted development of novel structurally distinct TKIs (Abdel-Aziz et al., 2015; Aziz et al., 2016). Among these second-generation TKIs, sunitinib, was designed as an oral small molecule ATP mimetic which competes with endogenous ATP for binding at the catalytic site of several tyrosine kinase receptors including vascular endothelial growth factor receptor (VEGFR), platelet-derived growth factor receptor (PDGFR), fms-like tyrosine kinase-3 receptor (FLT3) and stem cell factor receptor (c-kit) which are preferentially overexpressed in diverse types of cancer (Cella et al., 2015). In 2006, sunitinib became the first drug jointly approved by the FDA for treating both metastatic renal cell carcinoma (mRCC) and imatinib-resistant gastrointestinal stromal tumor(GIST) patients. Five years later, treatment of progressive pancreatic neuroendocrine tumors(pNET) was added to sunitinib indications. Since then, several clinical trials were initiated to evaluate its efficacy against diverse cancer types including those with limited therapeutic options as differentiated thyroid and invasive lower urothelial cancer. Nonetheless, intrinsic as well as acquired resistance to sunitinib rapidly emerged as a challenge restraining its clinical benefit (Table 1) (Adelaiye et al., 2014). In fact, almost one-third of sunitinib-treated patients are intrinsically resistant whereas the initially responders eventually relapse and develop progressive disease resulting in modest overall survival benefit (Stacchiotti et al., 2012; Adelaiye et al., 2014). Given the complexity of the target spectrum modulated by sunitinib, deeper understanding of the contribution of those targets to the sensitivity or resistance to sunitinib is cardinal for its optimal clinical use. Among chemoresistance mechanisms, other than mutation or amplification of drug targets, activation of prosurvival signaling pathways is a frequently exploited strategy by cancer cells to evade cell death and sustain their proliferation (Hammers et al., 2010; Shojaei et al., 2010).

**Table 1:** Clinical trials investigating the efficacy and safety/tolerability of sunitinib against different cancer types.

**Tumor type**	**Clinical status**	**Therapeutic combination**	**ClinicalTrials.gov identifier**	**Notes**
Relapsed or refractory esophageal or gastro-esophageal junction cancer.	Phase II	Monotherapy	NCT00702884	Sunitinib was well tolerated but only a subset of treated patients benefited [10 out of 25 (42%) had stable disease > 10 weeks] (Wu et al., 2015).
Extensive-stage small cell lung cancer.	Phase II	Sunitinib as a maintenance therapy following induction platinum + etoposide based therapy	NCT00616109	Sunitinib did not maintain disease stability following response to chemotherapy (only 4/16 [25%] patients had stable disease). Sunitinib was discontinued due to disease progression (50%), toxicity (31%), and patient request (19%) (Schneider et al., 2011).
Metastatic breast cancer.	Phase II	Monotherapy	NA	Sunitinib was modestly active in patients with heavily pretreated metastatic breast cancer (11% partial response and 5% stable disease). Most adverse effects were mild-to-moderate and managed with supportive treatment and/or dose modification (Burstein et al., 2008).
Refractory or relapsed small cell lung cancer.	Phase II	Monotherapy	NCT00620347	Partial tumor response was reported in 2 out of 23 patients, The median progression free survival was short and sunitinib was not tolerated in most patients did not tolerate sunitinib (Han et al., 2013).
Relapsed or refractory germ cell tumor (resistant to standard platinum-based chemotherapy)	Phase II	Monotherapy	NCT00453310	Sunitinib was well tolerated, but at standard doses, did not demonstrate significant activity in highly refractory germ cell tumor (no objective responses were found and all patients developed progressive disease within three cycles of sunitinib) (Feldman et al., 2010).
Local or metastatic papillary and non-clear cell renal cancer.	Phase II	Monotherapy	NCT00459875	Out of 22 evaluated patients, only one partial response was observed in unclassified metastatic renal cell carcinoma patient. No objective responses were found in patients with papillary metastatic renal cell carcinoma and non-clear cell histologies (Molina et al., 2013).
Non-clear cell renal cancer.	Phase II	Monotherapy	NCT00465179	Out of 55 analyzed enrolled patients, three had partial response, 29 and 23 had stable and progressive diseases respectively.
Cytokine refractory metastatic renal cell carcinoma.	Phase II	Monotherapy	NCT00077974	Sunitinib demonstrated efficacy and manageable adverse-event profile as a monotherapy in second-line therapy for patients with cytokine-refractory metastatic clear-cell RCC (Motzer et al., 2006).
Progressive metastatic transitional cell cancer of the urothelium.	Phase II	Monotherapy	NCT00397488	3 out of 71 patients had partial response. 29/71 (40.9%) had stable disease. Almost 55% (39/71) progressed.
Advanced prostate cancer.	Phase II	Monotherapy	NCT00299741	Sunitinib was well tolerated with modest benefit (Michaelson et al., 2009).
Metastatic colorectal cancer.	Phase II	In combination with capecitabine	NCT00961571	This study was terminated due to unanticipated side effects and futility.
Brain metastases caused by kidney cancer or melanoma.	Phase II	Monotherapy	NCT00462982	Out of the five patients who completed the study, three had stable disease and two progressed.
Imatinib resistant metastatic dermatofibrosarcoma protuberan.		Prior to sunitinib, patients could undertake other chemotherapy, radiotherapy and local surgery.	NA	Out of 30 imatinib-resistant patients, two had complete response (6.7%), 10 had partial response (33.3%), 12 had stable disease (40%) and 6 porgressed (20%). The progression free survival of complete response and partial response patients were 22 months and 20 months respectively. Hence, sunitinib therapy conferred good clinical efficacy and tolerated adverse effects as a new in imatinib resistant dermatofibrosarcoma protubern (Fu et al., 2015).
Metastatic mucosal or acral melanoma.	Phase II	Monotherapy	NCT00577382	Sunitinib was active against mucosal and acral melanoma that was independent of KIT mutation. Nevertheless, it was poorly tolerated, and with no prolonged responses (Buchbinder et al., 2015).
Recurrent, refractory, or progressive malignant glioma or ependymoma.	Phase II	Monotherapy	NCT01462695	Sunitinib was well tolerated in children and young adults with recurrent high- grade glioma or ependymoma. Sunitinib therapy significantly modulated plasma VEGFR2. Nevertheless, there were no sustained antitumor responses (Wetmore et al., 2016).
Metastatic, locally advanced, or locally recurrent sarcomas (non-gastrointestinal stromal tumor sarcoma).	Phase II	Monotherapy	NCT00474994	One patient achieved a confirmed partial response. 10 patients (20%) achieved stable disease for at least 16 weeks. There were no unexpected toxicities observed (George et al., 2009).
Metastatic pancreatic cancer.	Phase II	Monotherapy -maintenance therapy	NCT00967603	Results have not published yet.
Recurrent or inoperable meningiomas.	Phase II	Monotherapy	NCT00589784	Out of 35 analyzed patients with aggressive meningiomas, one had complete response, one had partial response, 25 had stable disease and eight progressed. The four patients with WHO grade I meningioma and hemangioblastoma treated with sunitinib progressed.
Advanced gastric cancer.	Phase II	Together with docetaxel	NCT01238055	Results have not published yet.
Inoperable liver cancer.	Phase II	Monotherapy	NCT00699374	Continuous daily sunitinib treatment (37.5 mg) was feasible and had moderate activity in patients with advanced hepatocellular carcinoma (Koeberle et al., 2010).
Myelodysplastic syndromes or chronic myelomonocytic leukemia.	Phase II	Monotherapy	NCT00451048	10 patients completed the study, but no statistical analysis is provided for the overall response Rate (complete response, partial response, or hematologic improvement).
Persistent or recurrent clear cell ovarian cancer.	Phase II	Monotherapy	NCT00979992	Out of 30 analyzed patients, the % of objective tumor response rate (complete and partial response) is 6.7.
Advanced urothelial carcinoma.	Phase II	Monotherapy	NCT00393796	Maintenance sunitinib did not appear to improve the 6-month progression rate. Open-label sunitinib had only modest activity (Grivas et al., 2014).

*NA: Not available*.

## Biphasic modulation of autophagy by sunitinib

The role of autophagy in tumorigenesis has been somewhat controversial (Yang et al., 2011; Abdel-Aziz et al., 2015). In addition to the well-established pro-survival functions during nutrient-deprivation, evidence suggests cytotoxic effect of excessive autophagy triggered by conditions other than starvation. Overwhelming autophagy induction may contribute to cell death through digesting essential cellular macromolecules and organelles and hence, is classified as programmed cell death type II (Maiuri et al., 2007). In line with this controversy, autophagy modulation has been suggested to contribute to both resistance and cytotoxicity of several chemotherapeutics (Yang et al., 2011; Abdel-Aziz et al., 2015). Throughout the last few years, controversial regulatory effects of sunitinib on autophagy have been reported. For instance, sunitinib activated autophagy in RCC, phaeochromocytoma, thyroid and breast cancer (Lin et al., 2012; Ikeda et al., 2013; Abdel-Aziz et al., 2014a,b). In contrast, others have reported negative regulatory effects on autophagic flux in human urinary bladder and medullary thyroid cancer (Santoni et al., 2013; Lopergolo et al., 2014).

**Figure 1:**
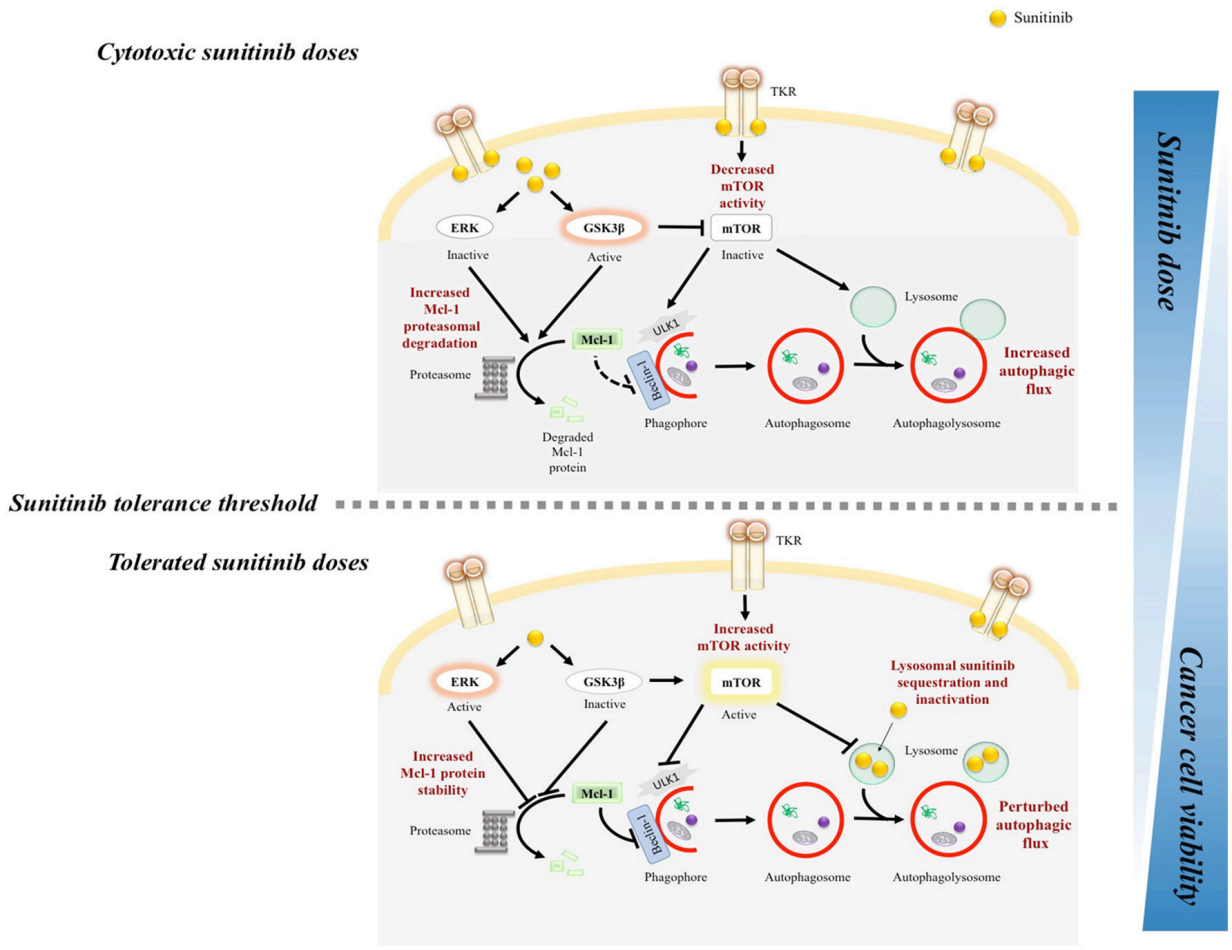
Mechanistic illustration of sunitinib dose-range dependent biphasic regulation of autophagy in cancer cells. TKR, tyrosine kinase receptor. Autophagy was inhibited in cancer cells that resist the anticancer activity of clinically relevant sunitinib levels. In response to tolerated doses, sunitinib increased ERK, and GSK3β phosphorylation which increased Mcl-1 stability, and mTOR activity. In addition, given its lysomotropic property, sunitinib resistance has been linked to its lysosomal sequestration, and hence, inactivation. In contrast, escalating sunitinib dose or pharmacologically targeting Mcl-1/mTOR restored cancer cell responsiveness/sensitivity to sunitinib.

In our recently published study, we aimed to systematically address this discrepancy (Elgendy et al., 2016). We primarily screened the anticancer efficacy of a broad range of sunitinib concentrations against a panel of human cancer cell lines representative of diverse types; renal cell carcinoma, pancreatic neuroendocrine tumor, colorectal cancer and osteosarcoma (Elgendy et al., 2016). Each cancer cell type had its unique “*sunitinib-tolerance threshold*” beyond which its viability was dramatically compromised. Strikingly, most cancer cell types tolerated sunitinib levels analogous to that of treated cancer patients. In accordance with our preclinical findings where higher sunitinib doses were cytotoxic, cancer patients who primarily progressed in response to standard sunitinib doses and during “sunitinib-off” period were resensitized by escalating sunitinib dose (Stacchiotti et al., 2012; Mitchell et al., 2015). Intriguingly, sunitinib tuned the activity of the autophagic machinery in a *biphasic pattern*. In response to tolerated doses—regardless of cancer type—sunitinib inhibited autophagy as evidenced by decreased GFP-LC3 puncta, LC3II/I ratio and increased p62/SQTSM1 levels. In contrast, autophagy was triggered in cancer cells challenged with cytotoxic sunitinib doses (Elgendy et al., 2016) (Figure 1).

Below, we review the differential modulatory effects of sunitinib on autophagy and their link to sunitinib resistance.

### Biphasic mTORC1 and Mcl-1 modulation mediates dual regulation of autophagy by sunitinib

Autophagy is regulated by a complex network of molecular switches, among which mammalian target of rapamycin (mTOR) is a central player (Yang et al., 2011). mTOR exists in two distinct complexes, mTORC1 and mTORC2. Unlike the relatively unexplored role of mTORC2 in cancer biology, mTORC1 is a nutrient sensitive sensor that orchestrates cell metabolism, cell cycle progression and autophagy (Yang et al., 2011). mTORC1 represses the latter at both initiation and degradation stages via inhibiting ULK1 complex and lysosomal function respectively (Elgendy et al., 2014; Puertollano, 2014). Furthermore, antiapoptotic B-cell lymphoma 2 (Bcl-2) family members such as Bcl-2, myeloid cell leukemia 1 (Mcl-1), and B-cell lymphoma-X large (Bcl-xL) - through their direct and indirect interactions with Beclin-1/Atg6 - have emerged as autophagy regulators which mediate the regulatory crosstalks between apoptosis and autophagy (Elgendy et al., 2014).

Intriguingly, in our study, cancer cells responded to subcytotoxic sunitinib doses by activating mTORC1 and increasing Mcl-1 protein levels (Elgendy et al., 2016) (Figure 1). Importantly, increased mTORC1 activity and Mcl-1 level represented a pro-survival cellular response to the mild to moderate stress triggered by these sunitinib doses since inhibiting mTORC1 or depleting Mcl-1 sensitized cancer cells to tolerated doses (Elgendy et al., 2016). Notably, mTORC1 activity and Mcl-1 levels were found to be higher in sunitinib-resistant compared to parental melanoma cells further confirming their role in mediating sunitinib resistance (Elgendy et al., 2016). Finally, analysis of pNET and RCC samples obtained from sunitinib-resistant patients showed a significant correlation between post-sunitinib increase in Mcl-1 levels and mTORC1 activity and resistance to treatment with sunitinib (Elgendy et al., 2016). In line with our observations, Makhov et al. has shown that deletion of PTEN (phosphatase and a tensin homolog deleted from chromosome 10), a negative regulator of PI3K/AKT/mTOR signaling, correlated with sunitinib resistance in renal and prostate cancer (Makhov et al., 2012). Conversely, cytotoxic doses dramatically diminished mTORC1 activity and Mcl-1 levels. In alignment with our findings, sunitinib was active against acute myeloid leukemias (AML) possessing activating PDGFR, FLT-3, and c-kit mutations through inhibiting mTOR and reducing Mcl-1 levels (Ikezoe et al., 2006; Teng et al., 2013).

Interestingly, in addition to genetic tools, our results showed that pharmacological mTOR and/or Mcl-1 inhibition using rapamycin and sorafenib respectively sensitized cancer cells to primarily tolerated doses of sunitinib (Elgendy et al., 2016). In accordance with our findings, Lin and colleagues demonstrated that while silencing Atg5 abrogated sunitinib cytotoxicity, everolimus and trehalose, mTOR-dependent and independent autophagy activators respectively, boosted the antiproliferative activity of sunitinib in medullary thyroid cancer cells (Lin et al., 2012). Furthermore, Atg5 silencing antagonized everolimus- and trehalose-triggered increase in sunitinib efficacy (Lin et al., 2012). Additionally, our data provide mechanistic rationale for the previously reported synergy between sunitinib and mTOR inhibitors identified through unbiased binary screening (Li et al., 2014).

### Sunitinib-induced ERK and GSK3β modulation lead to mTORC1 and Mcl-1 regulation

Deeper mechanistic analysis showed that the increase in Mcl-1 protein levels upon treatment with tolerated doses of sunitinib was associated with decreased Mcl-1 proteasomal degradation and enhanced stability, rather than modulation of gene expression (Elgendy et al., 2016). One distinct feature of Mcl-1 among other antiapoptotic Bcl-2 family members is its short half-life. This has been linked to its tight regulation by complex mechanisms on several levels including phosphorylation in its unique PEST region at Thr163 and Ser159 residues by extracellular signal regulated kinase (ERK) and glycogen synthase kinase 3β (GSK3β) respectively which in turn, diminishes and enhances the rate of Mcl-1 proteasomal degradation (Domina et al., 2004; Yan et al., 2017). Enhanced Mcl-1 stability in response to lower doses of sunitinib was the net result of both ERK activation and GSK3β inhibition (Elgendy et al., 2016). Conversely, higher “cytotoxic” doses led to opposite effects where ERK was inhibited and GSK3β was activated, resulting in Mcl-1 destabilization (Elgendy et al., 2016). Furthermore, dual modulation of GSK3β activity by lower and higher doses of sunitinib also mediated the differential effect on mTORC1 activity (Figure 1).

### Regulatory effects of sunitinib on AXL and MET signaling modulate ERK and GSK3β activity

Induced AXL and MET signaling which in turn increased ERK and GSK3β phosphorylation has been linked to sunitinib resistance in RCC (Zhou et al., 2016). Intriguingly, Qu et al. have lately identified a novel long non-coding RNA called lncARSR (lncRNA Activated in RCC with Sunitinib Resistance) which promoted sunitinib resistance via acting as a competing endogenous RNA. Indeed, IncARSR through sequestering miR-34 and miR-449 upregulated AXL/c-MET expression and activated ERK, STAT3 and AKT signaling. In a positive regulatory feedback loop, activated AKT boosted IncARSR expression. Strikingly, sunitinib-resistant RCC cells secreted IncARSR via exosomes which reached out to sunitinib sensitive cells and transformed them into resistant ones (Rna et al., 2016). Sunitinib-induced ERK activation secondary to increased EGFR phosphorylation was also linked to sunitinib resistance mediated by epithelial-to-mesenchymal transition (EMT) in mRCC cells (Mizumoto et al., 2015). In addition, sphingosine kinase-1 (SK1)-mediated ERK activation was triggered in sunitinib-resistant RCC cell lines which were resensitized to sunitinib using SK1 and ERK pharmacological inhibitors (Gao and Deng, 2014). Ras/Raf activating mutations and subsequent constitutive MEK/ERK activation has also been speculated to contribute to sunitinib resistance in thyroid carcinoma cells (Piscazzi et al., 2012). Moreover, sunitinib-induced apoptosis in medulloblastoma was associated with GSK3β and mTOR dephosphorylation (Yang et al., 2010).

### Lysosomal sequestration of sunitinib impedes autophagic flux

mRCC resistance to sunitinib was reported to be secondary to impedance of autophagy at its termination stage. Sunitinib neutralized the acidic lysosomal pH and hence, inactivated one of the major lysosome-associated proteases, cathepsin B (Giuliano et al., 2015). In light of the observations illustrating the tightly regulated crosstalk between lysosomes and mTORC1 where active mTORC1 negatively impacted lysosomal biogenesis, function and autophagosomes-lysosomes fusion (Puertollano, 2014), we postulate that subcytotoxic sunitinib doses-induced mTOR activation could also contribute to lysosomal dysfunction associated with the “chemoresistance phenotype.” Accordingly, it has been proposed that increasing sunitinib availability at its target TKRs via preventing its lysosomal trapping using agents which increase lysosomal membrane permeability could resensitize mRCC to sunitinib.

### Modulatory effects of sunitinib on ATP-binding cassette (ABC) transporters and autophagy

The role of multidrug resistance proteins has been linked to chemoresistance (Giuliano et al., 2015). Indeed, sunitinib increased ABCB1 expression favoring further lysosomal accumulation of sunitinib and its cellular efflux. Inhibiting this transporter by elacridar restored sunitinib senstivity in resistant mRCC (Giuliano et al., 2015; Sato et al., 2015). Sunitinib also increased ABCG2 levels in RCC cells (Sato et al., 2015) and glioma xenografts (Shojaei et al., 2010). Such increment is thought to compensate for the inhibitory effects of sunitinib on ABCG2 function (Dai et al., 2009; Shukla et al., 2009). While targeting ABC transporters might be useful in reversing sunitinib resistance, attention should be directed toward the bioavailability of co-administered drugs (Shukla et al., 2009; Sato et al., 2015). Interestingly, ABCG2 has also been shown to modulate autophagy (Ding et al., 2016). Compared to parental cells, ABCG2 overexpression—which was associated with boosted autophagy—rendered cancer cells more rendered cancer cells more resistant to stress inducers. Consistently, knocking down ABCG2 inhibited autophagy (Ding et al., 2016). Yet, it is still unknown whether modulating ABC transporters function/expression by sunitinib mediates its regulatory effects on autophagy and cancer cell sensitivity/resistance to sunitinib.

### Antiangiogenic effects of sunitinib, tumor microenvironment, and autophagy

As tumors progress, their oxygen and nutrients demands substantially outweigh their supply. To address these needs, “angiogenesis” is switched on generating tumor-associated neovasculature. Within this context, sunitinib emerged as an effective antiangiogenic agent via inhibiting VEGFR, PDGFR, and c-KIT, which was then indicated for treating tumors with high angiogenic burden as mRCC. Although an interrupted sunitinib treatment schedule of 4-weeks ON/2-weeks OFF was adopted to reduce its side effects, rapid tumor regrowth and metastasis considerably ensued during the 2-week break period (Ebos et al., 2009; Griffioen et al., 2012). While the underlying mechanisms remain elusive, microenvironmental changes are speculated to condition/acclimatize body organs to be more permissive to tumor extravasation by acting as “*premetastatic niche*” (Loges et al., 2009). Anti-angiogenic therapy—via triggering hypoxia—might induce autophagy in both tumor cells and microenvironment. While activating autophagy in cancer cells might retard their proliferation, stromal cancer-associated fibroblasts autodigest themselves into basic nutrients for epithelial cancer cells. This host-parasitic like paradigm is known as “*Battery-Operated Tumor Growth*” or “*The Autophagic Tumor Stroma Model of Cancer Cell Metabolism*” (Martinez-outschoorn et al., 2010). Cancer-associated cachexia is also hypothesized to start as local stromal autophagy, and then disseminates systemically. Consistently, increased metabolic rate of cachexic cancer patients is restored to their normal levels following surgical tumor excision (Martinez-outschoorn et al., 2010). Furthermore, sunitinib resistance is linked to persistence of intratumor myeloid derived suppressor cells (MDSCs) with dominating subset of neutrophilic population that produces proangiogenic MMP9, MMP8, and IL-8 (Finke et al., 2011). Activation of proangiogenic hepatocyte growth factor/c-Met signaling pathway also contributed to sunitinib resistance which was reversed using c-Met inhibitor (Shojaei et al., 2010). Notably, inhibiting c-Met signaling induced autophagic cell death in glioblastoma (Liu et al., 2013). Collectively, these divergent effects of antiangiogenics on primary tumor and its microenvironment necessitates elemental consideration of prominent issues while administering anti-VEGF therapy including; relative benefit-to-risk of “continuous vs. intermittent” treatment schedules, optimal dose, duration of treatment and tumor stage. This also emphasizes the importance of combination therapy as a possible approach to abrogate resistance to anti-VEGF therapy.

### Mitophagy, effects of sunitinib on mitochondrial structure/function and apoptosis

Mitophagy (or selective autophagic degradation of mitochondria) has been linked to tumorigenesis (Hjelmeland and Zhang, 2016). One of the key mitophagy mediators, Parkin, putative tumor suppressor gene, translocates to the mitochondria secondary to loss of mitochondrial membrane potential, ubiquitinating mitochondrial proteins and recruiting p62-LC3 and autophagosomes to the mitochondria (Hjelmeland and Zhang, 2016). Melatonin synergistically sensitized human heptocellular carcinoma cells to sorafenib through its pro-oxidant capacity and activating mitophagy (Prieto-domínguez et al., 2016). In fact, oxidative stress and hypoxia are fundamental autophagy inducers (Kulikov et al., 2017). Hypoxia - via hypoxia inducible factor 1α (HIF1α)- induced the expression of mitophagy receptors; BCL2/adenovirus E1B 19 kDa interacting protein 3 (BNIP3) and its homolog NIX (Kulikov et al., 2017). Though sunitinib blocked HIF1α translation and hence, repressed the expression of its downstream target genes in colorectal and renal carcinoma cells (Shin et al., 2010). Severe mitochondrial structural abnormalities was also reported in the heart of a patient with sunitinib-induced heart failure (Kerkela et al., 2008). This has been linked to its off-target inhibitory effect on AMPK (Kerkela et al., 2008). Sunitinib also triggered mitochondrial damage, cytochrome C release, caspase 9 activation and apoptotic cell death in cardiomyocytes *in vitro* and *in vivo* (Chu et al., 2007). Sunitinib increased both death receptor and mitochondrial-dependent apoptosis in AML cells (Teng et al., 2013). Nonetheless, it still remains to be elucidated whether sunitinib modulates mitophagy and therapeutic intervention with mitophagy could sensitize cancer cells to sunitinib.

### Linking the modulatory effects of sunitinib on autophagy to genomic instability

Dysfunctional autophagy has been connected to increased genomic instability. Intriguingly, sunitinib-induced increased autophagic flux concurred with increased micronuclei, diagnostic marker of nuclear instability, in human RCC (Yan et al., 2017). Nuclear LC3, phosphorylated Ulk1 and p62 interacted with Rad51 or PARP-1, which are both engaged in maintaining genomic stability (Yan et al., 2017). Sunitinib was incapable of accumulating micronuclei in p62/LC3-depleted cells. Depleting Rad51/PARP-1 abolished sunitinib-induced autophagy (Yan et al., 2017). Since p62 acts as the connecting bridge between ubiquitin and autophagic machineries, both systems are speculated to coordinate genomic stability. Despite being a marker of DNA damage, γ-H2AX actively participates in DNA repair. γ-H2AX and PARP-1/Rad51 interaction was disrupted following p62 depletion (Yan et al., 2017). Although sunitinib elevated γ-H2AX level, it decreased Rad51 expression which is essential for homologous recombination repair, Accordingly, while sunitinib induced acute DNA damage may lead to cancer cell death, it might also trigger non-homologous end joint DNA repair mechanisms. Collectively, these findings proposed a mechanistic link between the modulatory effects of sunitinib on autophagy and nuclear instability.

## Adverse effects of sunitinib and autophagy

Clinical trials and post-marketing surveillance have reported that sunitinib use is associated with several adverse effects including cardiac failure and cognitive impairment. In this regards, it has been shown that sunitinib-induced autophagic cell death contributed to its cardiotoxicity (Zhao et al., 2010). Impeded autophagic flux has been associated with sunitinib-induced chemobrain (chemotherapy-related cognitive impairment) (Abdel-Aziz et al., 2016). As our data strongly suggest a potential therapeutic synergy of a combination of sunitinib with Mcl-1/mTORC1 inhibitors such as sorafenib and rapalogues which are known to induce autophagy, this could be of crucial clinical relevance especially concerning the toxicity of such combination. Attempts to combine other drugs with sunitinib have thus been so far unsuccessful, largely due to toxicity. However, our *in vivo* results demonstrated a strong synergy on tumor xenograft growth of such combinations at doses lower that those used clinically with favorable tolerability/no sign of toxicity.

## Translating preclinical findings to bedside

### Novel predictive markers of sunitinib responsiveness

Canonical clinicopathological evaluation is unable to predict the therapeutic response to sunitinib-treated cancer patients. Identification of novel molecular prognostic markers is therefore urgently needed. Based on the present findings, immunohistochemical assessment of ribosomal S6 phosphorylation (as readout of mTORC1 activity) and Mcl-1 protein levels could serve as markers that predict sunitinib response. Additionally, elevated IncARSR levels in pre-treatment RCC patients significantly correlated with poor sunitinib response. In contrast, low IncARSR levels conferred improved progression-free survival and favorable prognosis following sunitinib therapy (Rna et al., 2016). Notably, IncARSR levels were remarkably increased in patients who regressed and relapsed post-sunitinib therapy compared with pre-therapy levels. Hence, IncARSR is proposed as an independent predictor for sunitinib response in RCC patients (Rna et al., 2016).

### Emerging therapeutic modalities to overcome sunitinib resistance

Additionally, the present findings provide a rationale for the lack of cytotoxic effects of clinically relevant doses of sunitinib, and suggest novel strategies -in addition to its anti-angiogenic effects- to directly induce tumor cell death, and overcome sunitinib resistance;

(i) Ideally, though not easily achievable in clinical practice, tailoring sunitinib dose per each patient based on their response should select patients that need escalation of sunitinib dose to reach cytotoxic effects at tolerable doses. Rovithi et al. showed that an alternating schedule of high sunitinib efficiently impaired tumor growth *in vivo* and maintained significantly higher plasma and intratumoral sunitinib levels compared to the standard, daily regimen (Rovithi et al., 2016). Accordingly a phase I clinical trial (NCT02058901) was initiated to investigate the safety, tolerability and efficacy of intermittent, high dose sunitinib schedules (300 mg sunitinib, administered once weekly) in patients with advanced solid tumors, with preliminary indications of prolonged disease stabilization and tolerability in a heavily pretreated group of patients (Rovithi et al., 2016).

(ii) Alternatively, pharmacological targeting of Mcl-1 and mTOR presents a promising strategy in combating/reversing sunitinib resistance. It is worth mentioning that FDA has already approved sequential treatment of sunitinib-resistant mRCC patients with everolimus, mTOR inhibitor. In addition, a phase I clinical trial aiming at determining the highest tolerable dose of “sunitinib and temsirolimus (mTOR inhibitor)” combination that could be administered to mRCC patients has lately been completed. There are also parallel clinical trials testing the efficacy of sorafenib in treating sunitinib-resistant mRCC and GIST patients. Yet, the clinical outcome of combining sunitinib and Mcl-1 and/or mTOR inhibitor in sunitinib-resistant patients remains to be investigated. Consistent with the emerging role of MET and AXL signaling in mediating sunitinib resistance, combining a small molecule inhibitior of both MET and AXL, BMS777607, with sunitinib both suppressed the growth of sunitinib-resistant RCC xenografts and prevented the emergence of sunitinib resistance (Rna et al., 2016). In support with this notion, a randomized Phase III clinical trial has further confirmed the clinical value of cabozantinib, a multityrosine kinase inhibitor targeting MET, VEGFR, and AXL in RCC patients who had progressed following VEGFR inhibitor therapy including sunitinib (Géczi et al., 2015).

## Open questions

In a broader scope beyond sunitinib, accumulating evidence reported compensatory activation of PI3K/AKT/mTOR signaling pathway and/or heightened Mcl-1 expression in diverse cancer types resistant to other TKIs including imatinib, dasatinib, erlotinib, and gefitinib (Burchert et al., 2005; Wu et al., 2016). This raises questions:

- Whether activation of mTOR/Mcl-1 signaling is a common adaptive resistance mechanism exploited by cancer cells—regardless of their origin and type—to evade the anticancer activity of TKIs.
- And accordingly, whether pharmacological targeting of mTOR/Mcl-1 could be adopted in TKIs-resistant patients. In line with this, two Phase I/II clinical trials assessing the safety and efficacy of combining imatinib and everolimus in treating imatinib-resistant CML and GIST patients have been launched.

## Concluding remarks

Sunitinib resistance in patients correlated with reinforced negative regulation of autophagy (increased Mcl-1 stability and mTORC1 activity) and lysosomal dysfunction (owing to sunitinib sequestration and hence inactivation). Both events acted as pro-survival adaptive responses that compromised the anticancer activity of sunitinib. Conversely, cytotoxic sunitinib levels destabilized Mcl-1, inhibited mTORC1 and activated autophagy. Hence, this may mechanistically resolve the previously described discrepancy in terms of “ON/OFF” autophagy regulation by sunitinib. Gaining deeper mechanistic insights into sunitinib resistance would provide better prognostic biomarkers that could guide clinicians toward patient stratification and more individualized therapy. Importantly, this would offer more innovative therapeutic strategies/approaches to overcome sunitinib resistance which could ultimately be translated to a better clinical outcome.

## Author contributions

AKA conceived and wrote the manuscript. ABA, SS, SM, and ME revised the manuscript. SM and ME reviewed the outline and content of the manuscript. All authors have read and approved the submitted manuscript for publication.

### Conflict of interest statement

The authors declare that the research was conducted in the absence of any commercial or financial relationships that could be construed as a potential conflict of interest.

## Abbreviations

ERK: extracellular regulated kinase
EMT: epithelial to mesenchymal transition
FLT3: fms-like tyrosine kinase-3 receptor
GSK3β: glycogen synthase kinase 3β
GIST: gastrointestinal stromal tumor
HIF1α: hypoxia inducible factor 1α
lncARSR: lncRNA Activated in RCC with Sunitinib Resistance
MDSCs: myeloid derived suppressor cells
mRCC: metastatic renal cell carcinoma
mTOR: mammalian target of rapamycin
PDGFR: platelet derived growth factor receptor
pNET: progressive pancreatic neuroendocrine tumor
TKI: tyrosine kinase inhibitor
VEGFR: vascular endothelial growth factor receptor.
