# Corrigendum: From Resistance to Sensitivity: Insights and Implications of Biphasic Modulation of Autophagy by Sunitinib

**DOI:** 10.3389/fphar.2018.00101

**Published:** 2018-02-12

**Authors:** Amal Kamal Abdel-Aziz, Ashraf B. Abdel-Naim, Samia Shouman, Saverio Minucci, Mohamed Elgendy

**Affiliations:** ^1^Department of Experimental Oncology, European Institute of Oncology, Milan, Italy; ^2^Department of Pharmacology and Toxicology, Faculty of Pharmacy, Ain Shams University, Cairo, Egypt; ^3^Cancer Biology Department, National Cancer Institute, Cairo University, Cairo, Egypt; ^4^Department of Biosciences, University of Milan, Milan, Italy; ^5^Max F. Perutz Laboratories, Department of Microbiology and Immunobiology, University of Vienna, Vienna, Austria

**Keywords:** autophagy, cancer, Mcl-1, mTOR, resistance, Sunitinib

In the original article, acknowledgment of funding by the Mahlke-Obermann Stiftung and the European Union's Seventh Framework Programme for research, technological development and demonstration under grant agreement no. 609431/INDICAR (Interdisciplinary Cancer Research) Postdoctoral Fellowship Programme of the University of Vienna to ME was missing. The authors apologize for this oversight. This error does not change the scientific conclusions of the article in any way.

The original article has been updated.

## Conflict of interest statement

The authors declare that the research was conducted in the absence of any commercial or financial relationships that could be construed as a potential conflict of interest.

